# Vitamin D Deficiency and Its Clinical Results in Preeclamptic Mothers and Their Babies

**DOI:** 10.7759/cureus.23519

**Published:** 2022-03-26

**Authors:** Ömer Tammo, Süleyman Yıldız

**Affiliations:** 1 Obstetrics and Gynaecology, Mardin State Hospital, Mardin, TUR; 2 Pediatrics and Child Health, Mardin Derik State Hospital, Mardin, TUR

**Keywords:** preeclamptic mothers newborns, preeclamptic mothers babies, preeclamptic mothers, hypertension in pregnancy, 25 (oh) vitamin d, newborn health risk, vitamin d supplementation, umbilical cord blood, preeclampsia, vitamin-d deficiency

## Abstract

Introduction: Vitamin D deficiency during pregnancy may lead to many health problems by negatively affecting the metabolism of the newborn and the mother, such as infantile rickets, poor fetal and neonatal growth and development, gestational diabetes, and preeclampsia. We aimed to investigate the levels and clinical results of vitamin D in preeclamptic mothers and their babies.

Methods: The study group consisted of 42 preeclamptic mothers, and their babies diagnosed with preeclampsia according to the International Society for the Study of Hypertension in Pregnancy (ISSHP) criteria, while the control group consisted of 49 healthy mothers and babies with similar gestational age and birth weight. All pregnant women participating in the study were routinely taking 1200 IU of vitamin D3 daily supplements. The cord blood vitamin D level of both groups of newborns was measured and the results were statistically compared.

Results: The birth week, weight, and height averages and APGAR score averages measured at the first minutes of the babies in the study group (preeclamptic mother babies) were statistically significantly lower than those of the babies in the control group (babies of healthy mothers) (p=0.001, p=0.001, p<0.001, p=0.004, respectively). Vitamin D and calcium levels of the mothers in the study group were lower than those of the mothers in the control group. When the infants were examined, only the level of vitamin D was statistically significantly lower in infants in the patient group (p<0.001, p<0.001, p=0.001, respectively).

Conclusion: There is consistent evidence of an association between low vitamin D concentrations and adverse preeclampsia outcomes. Since vitamin D deficiency is more common in preeclamptic mothers and their infants, higher-dose vitamin D supplementation than routine may be recommended to the patients.

## Introduction

Vitamin D deficiency in pregnant women and their babies is an important health problem with severe consequences by negatively affecting the metabolism of both [1], such as infantile rickets, poor fetal and neonatal growth and development, gestational diabetes, and preeclampsia [2].

Preeclampsia is a multisystemic pregnancy disease seen approximately in 4% of pregnancies and has adverse maternal-fetal and neonatal effects [3]. Although the definition of preeclampsia differs between countries and textbooks, many of them use the definition provided by the International Society for the Study of Hypertension in Pregnancy (ISSHP), which is used worldwide. The ISSHP defines preeclampsia as the presence of new-onset hypertension and proteinuria or other end-organ damage after 20 weeks of gestation [4].

According to many studies in the literature, it has been stated that a higher rate of preeclampsia can be seen in pregnant women with lower vitamin D levels. It is recommended to start vitamin D supplementation regularly for pregnant women with preeclampsia risk [5]. In line with the recommendations of the Ministry of Health in Turkey, pregnant women take 1200 IU of vitamin D3 supplements daily from the 12th week of pregnancy to the sixth month after delivery [6].

The frequency of preeclampsia is relatively high in our region (Mardin, Turkey), where we conducted this study. Thus, in this study, we aimed to investigate the levels and clinical results of vitamin D in preeclamptic mothers and their babies.

## Materials and methods

This prospective study consisted of 42 preeclamptic mothers and their babies diagnosed with preeclampsia according to the ISSHP criteria, while the control group consisted of 49 healthy mothers and babies with similar gestational age and birth weight. This study was conducted between December 2020 and March 2021 in Mardin Derik State Hospital, Turkey. As the Ministry of Health recommended in Turkey, all pregnant women participating in the present study were routinely taking 1200 IU of vitamin D3 daily supplements from the 12th week. Pregnant women with another chronic or psychological disorder, including chronic hypertension, and newborns with chronic diseases, were not included in the study group. Local ethics committee approval was obtained for the research (Approval number: 37201737-806.02). The participating families were informed about the study parameters and they provided written consent in accordance with the Declaration of Helsinki.

In our study, vitamin D deficiency was defined as 25(OH)D level <20 ng/mL (50 nmol/L), while vitamin D insufficiency was defined as 25(OH)D level between 21 and 29 ng/mL (525-725 nmol/L) [7].

Collection of blood samples

Blood samples (2 mL) were taken as two different samples, from the mother and the baby. To determine 25(OH)D levels, blood samples were taken by experienced nurses from the maternal upper extremity vein (median cubital or cephalic vein) just before birth, while in newborns, blood samples were taken from the umbilical vein just after birth. After the samples were centrifuged at 4000 rpm for 6 minutes, they were stored at -20 degrees Celsius until the day of analysis. Serum 25(OH)D concentrations were measured using a chemiluminescence assay using the LIAISON instrument with the Diasorin kit (Diasorin Inc., Stillwater, MN, USA).

Statistical analysis

SPSS 15.0 for Windows program (SPSS Inc., Chicago) was used for statistical analysis. Descriptive statistics were given as numbers and percentages for categorical variables and mean, standard deviation, minimum, maximum, and median for numerical variables. The rates in the groups were compared with the chi-square test. Comparisons of numerical variables between two independent groups were made using the Mann-Whitney U test since the normal distribution condition was not met. The relationships between numerical variables were analyzed by Spearman's correlation analysis since the parametric test condition was not met. Cutoff analysis was performed by receiver operating characteristic (ROC) curve analysis. The statistical alpha significance level was accepted as p ˂0.05.

## Results

When the results were analyzed regarding demographic and clinical characteristics, the mean gestational week of the study group (preeclamptic mothers) was statistically significantly lower than that of the control group (healthy mothers), while the mean age, systolic and diastolic blood pressure, and proteinuria were statistically significantly higher (age p=0.019, other comparisons p<0.001). The birth week, weight and height averages and APGAR score averages measured at the first minutes of the babies in the study group (preeclamptic mother babies) were statistically significantly lower than the babies in the control group (babies of healthy mothers) (p=0.001, p=0.001, p<0.001, p=0.004, respectively). There was no statistically significant difference between the groups regarding head circumference, fifth-minute APGAR score, and gender (p=0.887, p=0.620, p=0.897, respectively) (Table 1).

**Table 1:** Comparison of demographic and clinical characteristics between groups

		Study (Preeclamptic) Group		Control Group		
		Mean±SD	Min-Max (Median)	Mean±SD	Min-Max (Median)	p
Mother						
Age		27.7±5.4	26 (21-41)	25.2±4.9	23 (19-38)	0.019
Gestational week		37.5±1.5	38 (33-39)	38.6±0.7	39 (37-39)	<0.001
Systolic blood pressure (mmHg)		153.6±8.9	155 (140-175)	117.7±10.1	120 (95-153)	<0.001
Diastolic blood pressure (mmHg)		92.7±8.8	95 (75-110)	73.2±8.9	75 (60-95)	<0.001
Proteinuria (g/dL)		1.80±0.81	2 (1-3)	0.24±0.43	0 (0-1)	<0.001
Baby						
Weight (g)		3250.2±430.4	3100 (2500-3900)	3528.0±303.7	3640 (2980-4100)	0.001
Height (cm)		46.4±4.7	48 (34-52)	49.5±1.9	50 (42-52)	<0.001
Head circumference (cm)		37.3±2.0	37 (32-42)	37.3±1.5	37 (35-40)	0.887
1st minute APGAR		6.76±0.88	7 (5-8)	7.35±0.86	7 (5-9)	0.004
5th minute APGAR		9.26±0.70	9 (7-10)	9.37±0.53	9 (8-10)	0.620
Gender n (%)	Male	22 (52.4%)		25 (51.0%)		0.897
	Female	20 (47.6%)		24 (49.0%)		

Vitamin D and calcium levels of the mothers in the study group were lower than those of the mothers in the control group. When the infants were examined, only the level of vitamin D was statistically significantly lower in infants in the patient group (p<0.001, p<0.001, p=0.001, respectively) (Table 2).

**Table 2:** Vitamin D, calcium, magnesium, and phosphorus levels in the groups

	Study (Preeclamptic) Group		Control Group		
	Mean±SD	Min-Max (Median)	Mean±SD	Min-Max (Median)	p
Mother					
Vitamin D (ng/mL)	19.3±13.3	15 (5-55)	39.2±17.2	39 (1090)	<0.001
Calcium (mg/dL)	8.59±0.49	8.6 (7.9-10)	9.10±0.59	9.1 (8-10.5)	<0.001
Magnesium (mg/dL)	2.22±0.26	2.2 (1.8-2.7)	2.19±0.20	2.2 (1.9-2.6)	0.835
Phosphorus (mg/dL)	3.29±0.60	3.3 (2.1-4.1)	3.28±0.66	3.5 (2-4.1)	0.903
Baby					
Vitamin D (ng/mL)	23.4±13.7	20 (10-60)	36.5±13.7	33 (10-65)	0.001
Calcium (mg/dL)	8.94±0.63	8.8 (7.8-11)	9.06±0.48	9 (8.2-10)	0.150
Magnesium (mg/dL)	2.14±0.34	2 (1.2-2.9)	2.09±0.23	2 (1.5-2.8)	0.267
Phosphorus (mg/dL)	3.40±0.71	3.65 (2-4.5)	3.31±0.66	3.6 (2-4.1)	0.395

## Discussion

Today, it is stated that maternal vitamin D deficiency may be an independent risk factor for preeclampsia [8]. The findings in previous studies suggest that the effects of vitamin D levels on the immune system and given that infections that occur as a result of vitamin D deficiency play a role in the development of preeclampsia [9]. Thus, starting vitamin D supplementation from early pregnancy can prevent possible health problems for both mother and baby by preventing preeclampsia [10].

The risk of preeclampsia may increase, especially in cases where 25(OH)D vitamin falls below 10 ng/mL (severe deficiency). For example, in a study conducted in the USA, severe preeclampsia was up to five times higher in pregnant women with insufficient 25(OH)D levels at the 15-20th weeks of pregnancy than in the control group [11]. In another study in which 33 studies were reviewed, there was a significant relationship between lower vitamin D levels and preeclampsia [12]. For example, in Halhali et al.’s study, which examines the relationship between vitamin D deficiency and preeclampsia, it has been shown that every one standard deviation increase in vitamin D level during 24-26th gestational weeks may cause a 30% decrease in the risk of developing preeclampsia [13]. In our study, the mean vitamin D level of the mothers in the study (preeclampsia) group was 19.3 ng/mL, while the vitamin D level of the babies of these mothers was 23.4 ng/mL. When the mothers of the control group were examined, it was observed that the average vitamin D level of the mothers was 39.2 ng/mL, while the average vitamin D level of the babies of these mothers was 36.5 ng/mL. In conclusion, our study showed that preeclampsia could be seen even in cases with vitamin D insufficiency (25(OH)D level 21-29 ng/mL), let alone vitamin D deficiency (<10 ng/mL), and may lead to clinical consequences in infants.

In a study in which 3730 mothers from different ethnic groups were followed, it has been reported that the risk of lower birth weight and preterm birth is higher in those with a vitamin D level below 12 ng/mL in the early stages of pregnancy [14]. In our study, it was observed that the birth height and weight of the babies of preeclamptic mothers were lower than those of the control group.

In some studies, it is stated that birth weight and calcium levels are better in newborns of mothers who take calcium and vitamin D supplements during pregnancy. Dosing recommendations for women during pregnancy and lactation might be best directed toward ensuring that the neonate is vitamin D-sufficient and that this sufficiency is maintained during infancy and beyond [15]. In our study, although the calcium level of preeclamptic mothers with significantly lower vitamin D levels was within normal limits, the calcium level was statistically significantly lower than that in the control group. Again, although the vitamin D level was lower in babies of preeclamptic mothers, no significant difference was found in the blood calcium levels when compared to that in the control group.

There are different recommendations about how much vitamin D to take during pregnancy. The recommendation of the USA Endocrine Society emphasizes that the target for vitamin D level in pregnancy should be >30 ng/mL (>75 mmol/L) and that 1500-2000 IU of vitamin D per day would be appropriate to reach this level [16]. Perez-Lopez, in his evaluation in 2020, reported that the vitamin D level should be 40-60 ng/mL during pregnancy, and the need for vitamin D is 4000 IU/day to achieve these levels [17]. However, preeclamptic mothers who participated in our study regularly taking the recommended daily 1200 IU vitamin D supplement in our country but still having vitamin D deficiency suggests that the amount of supplements taken may not be sufficient. The American College of Obstetricians and Gynecologists (ACOG) states that a daily dose of 4000 IU can be increased to treat vitamin D deficiency during pregnancy [18].

Although the prospective design of our study is its important strength, the relatively small number of participants, the absence of different ethnic origins, the limited number of samples, and possible selection bias are the limitations of this study.

## Conclusions

Vitamin D deficiency is a critical health problem for pediatricians, obstetricians, and family physicians to be alert. There is consistent evidence of an association between low vitamin D concentrations and adverse preeclampsia outcomes. Since vitamin D deficiency is more common in preeclamptic mothers and their infants, higher-dose vitamin D supplementation than routine may be recommended to patients.
